# Neutron activation analysis in Mediterranean Archaeology: current applications and future perspectives

**DOI:** 10.1007/s12520-023-01728-1

**Published:** 2023-02-11

**Authors:** Kai Riehle, Erich Kistler, Birgit Öhlinger, Christian Heitz, David Ben-Shlomo, Reinhard Jung, Hans Mommsen, Johannes H. Sterba, Stefanos Gimatzidis, Alexander Fantalkin, Susanne Prillwitz, Anno Hein, Leonhard Geissler, Gunnar Lehmann, Jan Kindberg Jacobsen, Richard Posamentir, Udo Schlotzhauer

**Affiliations:** 1 Institut für Archäologien, Universität Innsbruck, Langer Weg 11, 6020 Innsbruck, Austria; 2 Institut für Klassische Archäologie, Universität Tübingen, Burgsteige 11, 72070 Tübingen, Germany; 3 Institute of Archaeology, Ariel University, 40700 Ariel, Israel; 4 Austrian Archaeological Institute, Austrian Academy of Sciences, 1020 Vienna, Austria; 5 Helmholtz-Institut für Strahlen- Und Kernphysik, University of Bonn, Nussallee 14–16, 53115 Bonn, Germany; 6 Center for Labelling and Isotope Production, TRIGA Center Atominstitut TU, Wien, Stadionallee 2, 1020 Vienna, Austria; 7 Institute of Archaeology, Tel Aviv University, Haim Levanon St. 30, 6997801 Tel Aviv, Israel; 8 Institut für Ur- und Frühgeschichte und Vorderasiatische Archäologie, University of Heidelberg, Sandgasse 7, 69117 Heidelberg, Germany; 9 Institute of Nanoscience and Nanotechnology, N.C.S.R. “Demokritos”, PO Box 60037, 15341 Aghia Paraskevi, Greece; 10 Tübingen, Germany; 11 Dept. of Bible, Archaeology and Ancient Near Eastern Studies, Ben-Gurion University, PO Box 653, 84105 Beer Sheva, Israel; 12 Danish Institute in Rome, Via Omero 18, 00196 Rome, Italy; 13 Eurasia Department, German Archaeological Institute, Im Dol 2-6, Haus II, 14195 Berlin, Germany

**Keywords:** NAA, Mediterranean Archaeology, Pottery studies, Provenance determination, Connectivity, Interdisciplinarity

## Abstract

This paper, jointly written by participants of a workshop held in 2021, argues for an increased recognition and application of neutron activation analysis (NAA) in the archaeology of the ancient Mediterranean. Discussing the methodological strengths and challenges, it highlights the great potential NAA has for collecting proxy data from ceramics in order to develop progressive concepts of archaeological research within and beyond the Mediterranean Bronze and Iron Age, pointing out opportunities to revisit long-held assumptions of scholarship and to refine visual/macroscopic provenance determinations of pottery. To take full advantage of NAA’s strengths toward a better understanding of the socioeconomic background of ceramics production, distribution, and consumption, the paper emphasises the need for both interdisciplinary collaboration and basic data publication requirements.

**Supplementary Information:**

The online version contains supplementary material available at 10.1007/s12520-023-01728-1.

## The importance of archaeometric provenance studies and the potential of NAA

The origin of pottery is fundamental to archaeological research on multiple levels. As the ‘plastic waste of antiquity,’ ceramics are found almost everywhere. Their classification as locally made or imported still serves as an important starting point for analysing the material expression of the formation and transformation of social groups in the past, both in spatial and temporal terms. This includes the propagation of ideas, technologies, goods, and people—in short, the transfer and/or mobility of material culture and its creators/carriers, human beings. However, in recent years, archaeometric studies and an increasing awareness of the pronounced connectivity of ancient societies, and the complexity of the expression of material culture, have demonstrated how poorly reliable postulates of provenance based on macroscopic features alone may be—and thus the cultural-historical interpretations linked to them. Therefore, the increased application of archaeometric analysis is urgently needed, not only to verify previous assumptions of origin and related cultural hypotheses but also to provide proxy data for the application of up-to-date concepts of social archaeology. We are not concerned with abandoning indispensable visual methods of classifying ceramics out of blind faith in technological progress. However, we are convinced that for a better understanding of the production, distribution, and use of ceramics, different but inextricably linked approaches should be applied.

Several archaeometric methods have been increasingly employed to tackle current complex issues, and they provide us with various approaches to establish the possible provenance of objects. However, we argue that to obtain reliable and comparable results, neutron activation analysis (NAA) is one of the most important archaeometric methods in the study of the ancient Mediterranean (and any other region), firstly because of its general technical features, which have been applied since the 1960s (e.g., Sayre 1963; Perlman & Asaro 1969; Harbottle 1976). These include precision, the large number of principal and trace elements measured, the relatively small amount of sample material required (approx. 80 mg), high sample throughput, advanced methods of statistical data evaluation (Beier & Mommsen 1994), and the possibility to use data of different laboratories for interlaboratory comparisons. For archaeology, these features also constitute the strength of NAA as an analytical tool for establishing centres of pottery production, the typological range of vessels manufactured on-site, and the destinations of their spatial negotiation, which can be determined with a great degree of certainty. This may lead to a re-examination and possible revision of the previous postulates regarding the origins of artefacts. Furthermore, the data obtained can also serve as a starting point for questions concerning economic relations, cultural contacts, spatial and social mobility, knowledge transfer, consumption, the formation of social groups, and the negotiation of identities. Such issues are highly relevant in current research on the ancient Mediterranean.

In accordance with Neff et al. (2006, 70), we will not promote one archaeometric approach at the expense of another. The choice of method for a particular project depends on many factors, though it must fit the questions being asked. This methodological principle concerns all scientific analyses of ceramics, also the ‘integrated’ one proposed by Tite (1999), a combination of chemical analysis and petrography, recently referred to as a standard procedure for archaeometric pottery studies (Ownby et al. 2022). Indeed, such an interdisciplinary multiscalar approach is mandatory when addressing technological issues of local ceramic production (e.g., Fragnoli et al. 2021). For provenancing, however, additional petrographic analyses are less useful, given the consideration of experimental uncertainties, dilution effects, and appropriate sample selection (Mommsen 2004). Rather, NAA is often relied upon for the localisation of petrographic paste groups (Fragnoli et al. 2021; Habicht-Mauche & Eckert 2021, 763). Nevertheless, petrography can provide important information to regionally constrain unlocalized elemental patterns, as noted below.

## Reflexive acknowledgements

The thoughts and ideas presented in this paper are based on recent studies and the many years of experience that we as archaeometrists and/or archaeologists have been using NAA in numerous investigations of Mediterranean Bronze- and Iron Age ceramics, especially as developed by Hans Mommsen at the Helmholtz-Institut für Strahlen- und Kernphysik, University of Bonn. It is not just purely methodological interest that started the discussion but rather a concern for the comparability of independently collected sample series linked to this method that virtually ‘forces’ a dialogue between the different archaeological disciplines and bridges artificial boundaries drawn by political − historical constellations of the modern era, which is especially important in light of an increasing awareness of distinct connectivity in the past. In a sense, then, it is not despite, but precisely because of the apparent variations in the chronological and spatial emphases studied by each of us that we share a common conviction—the need to determine the provenance of ancient pottery as proxy data for further in-depth questions about the uses of material culture and NAA’s exceptional role for this purpose.

**Fig. 1:**
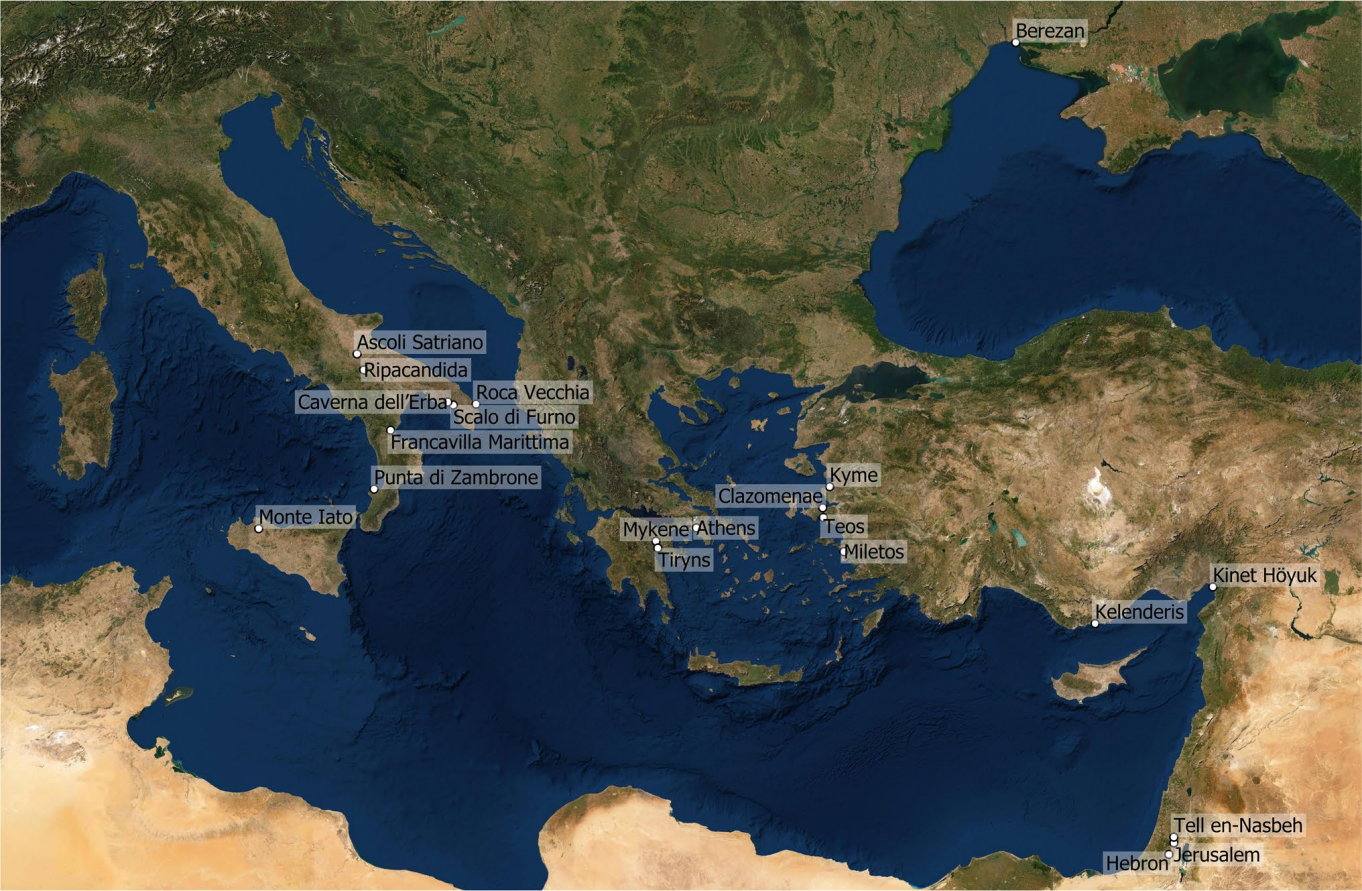
Sites mentioned in the text. All geographical coordinates are given online in Supplement 1

This paper is the result of the online workshop ‘Neutron activation analysis (NAA) and its relevance to the Archaeology of the ancient Mediterranean.’ Current state and perspectives of research were held in June 2021 by the Departments of Classical Archaeology at the Universities of Innsbruck and Tübingen, gathering together scholars of prehistoric Italic, Aegean, Near Eastern, Biblical, and Classical archaeology and archaeometrical specialists (Fig. 1). Since some of the studies mentioned have already been published recently or will be published soon, we have decided against issuing standard conference proceedings or reports. Rather, in a jointly written contribution, we would like to highlight the most important issues we have repeatedly confronted over the years, all of which were raised and discussed during the workshop, to underline the methodological strengths of NAA. This way, we further want to demonstrate the high potential NAA has for current approaches and questions relating to the study of the ancient Mediterranean and argue for a broader engagement with this long-established method. It can be applied to field projects and used to re-evaluate material stored in collections or exhibited in museums, stylistic studies of vase painting, and so on. NAA can help to hone and even change our views of the ancient Mediterranean and its societies. To emphasise these issues, the structure and content of this paper are presented in the form of a synopsis rather than a methodological review of issues relating to the provenance of pottery without denying limitations and difficulties of NAA and the interpretation of its results. With this contribution, however, we focus on NAA’s strengths—both as a stand-alone or an integrated approach, depending, as above highlighted, on the questions at hand. References in the text by author name (2021) are based on the oral presentations by the respective author at the workshop.

## Principles of NAA

**Fig. 2:**
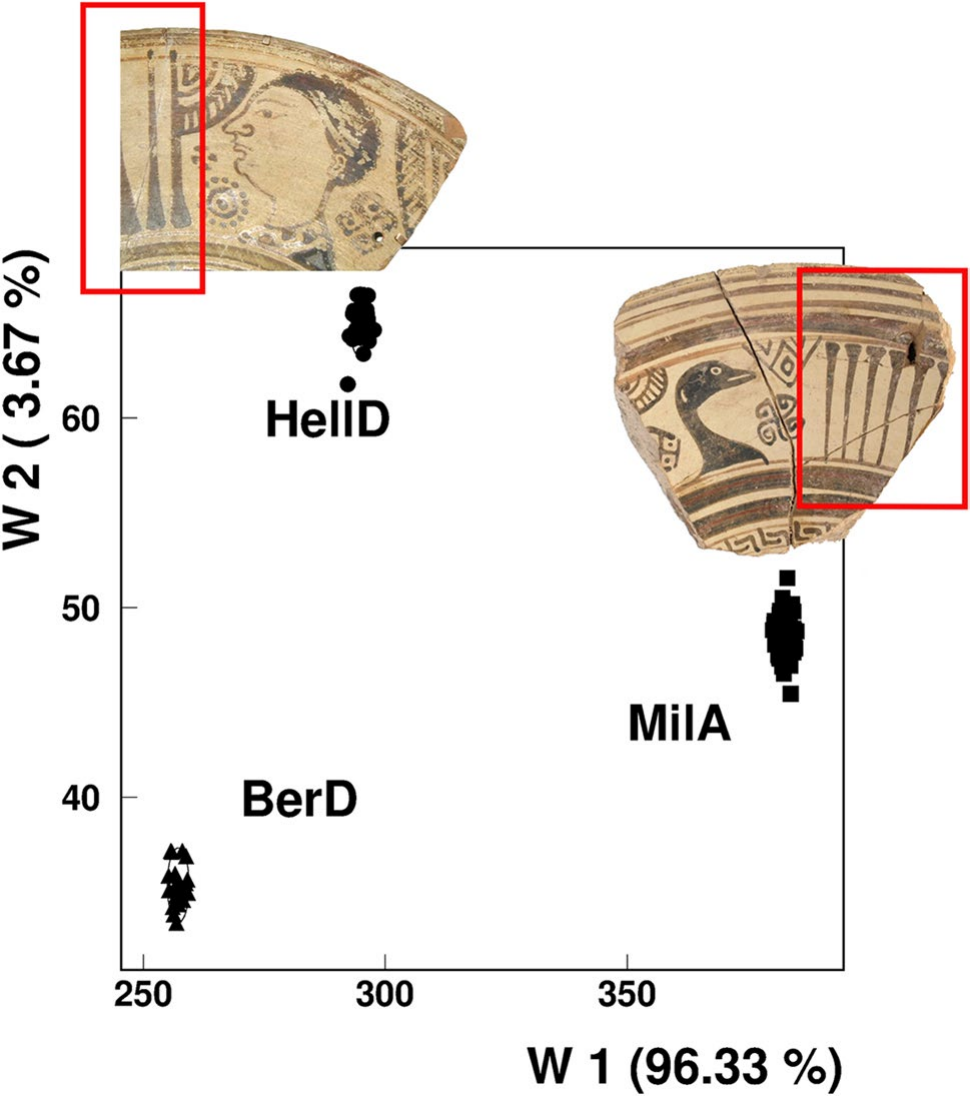
Result of a discriminant analysis (Bonn’s laboratory) of three chemical groups localized at Miletos (MilA), the Hellespont region (HellD), and Berezan (BerD). Given are the discriminant functions W1 and W2 describing 96% and 4% of the inter-group variance, respectively. In detail almost identical types of Eastern Aegean metope plates. Framed in red are the rays pointing inward on plates made at Miletus, while the rays on specimens made at the Hellespont workshops and found at Berezan pointing outward

At this point, it is worth recalling the principles of NAA (cf. Glascock & Neff 2003; Mommsen 2007; Minc & Sterba 2016). Neutron activation analysis is a minimally invasive method of determining the elemental concentration patterns in ceramics. The basic assumption is that vessels with the same elemental pattern, which varies to a limited extent, were made in the same place. A shared pattern of elemental concentration is often referred to as the chemical fingerprint of the corresponding wares (Fig. 2). The elemental composition of ceramics, in turn, depends on the raw clay used and the preparation of the clay before firing (Mommsen 2007; Hein & Kilikoglou 2020). This means that the elemental concentration pattern of the fired clay pastes does not necessarily have to match that of the raw clay source—which seems to occur only occasionally (e.g., Hein et al. 2004; Mommsen 2014). Thus, because it considers the ‘human factor’ of clay paste preparation, NAA is perfectly suited for tracing elemental compositions of pastes which often correspond to specific workshops or production locations. To localise an elemental pattern (or a fingerprint), reliable reference material must be sampled. Pottery waste and misfired pieces from ancient workshop contexts are of special importance in this regard because they were most probably made locally (i.e., not imported). Other vessels of a similar pattern, no matter where they were found, are most likely made from the same plastic part of a clay paste and therefore in the same workshop. The term *workshop* can be used to refer not only to a specific facility but also to an entire site. Conversely, a given site may also have multiple local patterns indicating several workshops or the use of different and even mixed pastes in one workshop (Kerschner 2002; Schwedt & Mommsen 2004; Fragnoli et al. 2021). In cases of a more household-based ceramic production, the variability of local elemental patterns may increase significantly (De Lucia et al. 2020).

Methods of statistical data analysis are crucial for the formation of chemical groups and the resulting archaeological evaluation of a sample series (Neff et al. 2006, 61; Mommsen 2007, 183–188; Minc & Sterba 2016, 439–441). The filter method developed at Bonn has proven to be a reliable tool over the last 30 years. It has allowed researchers to account for experimental uncertainties and/or shifts in element concentrations using a constant (dilution) factor (Mommsen et al. 1988; Beier and Mommsen 1994; Mommsen 2012). For instance, under laboratory conditions, Sterba et al. (2009) demonstrated that quartz-dominated tempers added to one and the same raw clay resulted in different groupings without data filters, which can lead to insufficient provenance assignments under field conditions. It was only by applying the filter method that the same plastic source material could be identified. To identify pastes that are blended with different ratios of non-plastic additives, but all originate from the same location, this is a key advantage, which should not, however, be mistaken for the existence of only one paste recipe. Thus, these methods make possible a more reliable assignment of a given sample to a specific chemical group and facilitate data-based comparisons with other sample series. In addition, the value of the filter factor itself can give additional information. For example, the concentration pattern of Late Bronze Age Attica is statistically identical to that of later periods but differs in its filter factor (Schöne-Denkinger & Mommsen 2009). Late Bronze Age vessels are made of a paste that is by a factor of about 10% more diluted than the paste used in classical times, indicating that in the surrounding of Athens potters of the Classic period applied other forms of paste preparation.

The opportunity to reconcile simple data is another methodological strength of NAA. Only by cross-referencing with previous and/or elsewhere conducted samplings can a single sample series be detached from its locality and placed in a broader spatial and temporal framework—even if the conception of a single project may already go beyond the local scale. Simply put, any new sampling series benefits from previous ones. The two largest databases for the ancient Mediterranean, currently holding about 20,000 individual datasets in total, have been created in Bonn and Athens and provide an inestimable important resource for future ceramic studies. If reliable calibration factors have been determined, NAA data from different laboratories can be compared, as Hein (2021) demonstrated by the ceraDAT database hosted at NCSR ‘Demokritos’ in Athens (see also Hein & Kilikoglou 2012).

## NAA applied to archaeology

The value of NAA for archaeological research, which has so far been described in mainly abstract terms, has been demonstrated time and again over the past decades. The benefits of NAA were also repeatedly highlighted in contributions to the 2021 workshop. These are presented according to their often interconnected and mutually reinforcing advantages, applications, and heuristic potential focusing on pottery provenances as proxy data for studying agency in the past. To examine further aspects, for example, more technological issues, such as paste recipes or firing temperatures, other methods must be involved.

### Determination and localisation of production sites/workshops

One of the basic tasks of NAA is to determine and localise elemental concentration patterns and thereby link the production of certain ceramics to specific locations. Its methodological value for this purpose has been impressively demonstrated previously, e.g., in the case of archaic Asia Minor. As a result of many years of systematic sampling, the most important production centres of fine ceramics and the types of wares produced on-site are well known: vessels of the South Ionian Wild Goat style (SWG) were almost exclusively produced in Miletos, and Clazomenae was a major production centre of the Northern Ionian Wild Goat style (NWG) as Teos for the famous Subgeometric bird bowls, to give just a few examples (summarised in Kerschner 2017, 108–112 and Geissler et al. 2022, 237–244). Many of the contributions held during the 2021 workshop presented new data. By identifying one local chemical fingerprint from Jerusalem, Ben-Shlomo (2021; 2019) offered evidence of the local production of Iron Age II tableware, cooking pots, and storage vessels and the extent of the distribution of these wares in the region. Just as for the localisation of the local chemical fingerprint of Hebron, wasters of a Roman period potter’s workshop were also used for Jerusalem, illustrating the potentially long duration of a clay paste recipe reflected by NAA. Meanwhile, Lehmann (2021) gave an insight into the spectrum of local pottery production between the twelfth and fourth centuries BCE in Kinet Höyuk in southeastern Cilicia, including Cypriot types from the Early Iron Age. Other workshop contributors reported the identification and localisation of chemical patterns in Greece, southern Italy, Sicily, and North Africa. As with the examples mentioned, however, this only served as a first but crucial step for more in-depth questions. For an even greater understanding of the nature of Mediterranean pottery production, we need to know what was produced, when, and where.

### Evaluation of previous postulates of origin

If the place of manufacture for certain ceramics is known, the previous postulates of origin—which are often based on macroscopic studies and/or the distribution of finds—are inevitably re-examined. Sometimes, this can lead to surprising results, as the aforementioned studies on the Eastern Aegean have demonstrated. For example, although NWG pottery was produced mainly in Clazomenae and Teos, similar vessels were made in the Aeolian region, possibly at Kyme (Kerschner 2017, 112f.). This has consequences for any new finds of such pottery in Aeolia, which no longer can automatically be attributed to Northern Ionian workshops. Gimatzidis (2021) expressed the need for similar caution in the case of other wares and regions. For instance, Central western Euboea is rightly considered an important production region for Early Iron Age pendent semicircle (PSC) skyphoi, as previous NAA research has demonstrated (Kerschner & Lemos 2014, 195–199). However, Gimatzidis’ examination of earlier and recent analytical and archaeological data has revealed that PSC skyphoi, including the oldest types, were produced by at least 12 workshops scattered across the entire Mediterranean. The new findings suggest that the local occurrence of PSC skyphoi need not inevitably be related to direct or indirect contacts with Euboea, as has often been assumed. Equally surprising were Geissler’s (2021) findings. In his study of Etruscan black-figure vase painting, he proved that at least one amphora of the so-called Northampton group was not produced in Etruria but in Athens, contrary to previous assumptions. However, other NAA results support assumptions about the origin of some pottery types that were based on conventional archaeological methods. Vessels featuring Attic figural painting, for instance, usually reveal a chemical pattern localised in Athens (Schöne-Denkinger & Mommsen 2009, 84f.). Similarly, a pattern localised in the northeastern Peloponnese can be traced in most sampled Mycenaean vessels found in the Levant, as Ben-Shlomo (2021) and Fantalkin (2021) elaborated in their survey on Israel (see also Maeir et al. 2009; Jung 2015; Zuckermann et al. 2020). The Milesian origin of the SWG pieces from several key Israeli sites has also been verified using NAA (Fantalkin 2021).

### Refining analysis based on visual characteristics

Chemical methods of analysis (including NAA) should never simply replace macroscopic classifications. This is neither quantitatively nor economically feasible nor desirable, either in the short or long run. However, from an archaeological point of view, NAA can be used as a tool to refine typological, stylistic, and iconographic pottery studies. Once again, an illustrative example can be found in the eastern Aegean: thanks to NAA, we now know that in most cases, the decorative rays of metope plates produced in Miletos are inward-orientated while the rays on plates of the very same type produced at the so-called Hellespont workshops point outwards (Fig. 2; Posamentir & Solovyov 2007, 195–198; Geissler et al. 2022, 241). This will be important in determining the provenance of future finds of this plate type. However, even for pottery where we have no information about findspots or context (e.g., in museums and collections), NAA again offers great potential due to the capability of database-driven comparisons of elemental concentration patterns. Thus, a stronger inclusion of archaeological fieldwork for studies based on pottery from museums, and vice versa, is possible. On the one hand, this can lead to the relocalisation of long-known vessels and of entire stylistic and/or typological groups, as Geissler (2021) demonstrated. He revealed that amphorae of the so-called Chalcidian and pseudo-Chalcidian vase painting of the sixth century BCE, which were previously assumed to have been made at different southern Italian sites, shared the same elemental concentration pattern, and were therefore most probably produced at the same place, though the exact location has yet to be established. In combination with stylistic analyses, NAA also offers the opportunity (as yet largely unrealized) to obtain a deeper understanding of the mobility of vase painters and their schools and the production and dissemination of certain iconographic themes (for an example of one of the few attempts, see Thorn and Glascock 2010). Schlotzhauer (2021) presented a quite different application of NAA of fundamental interest for museums and collections in particular. Once the chemical patterns of certain ceramics are reliably located, as is the case in archaic Ionia, NAA and stylistic analysis can help to distinguish modern forgeries from ancient originals. Since it is nearly impossible for forgers to imitate the chemical composition of a particular local clay paste from ancient times, this method has been used in juridical disputes concerning the illegal trade in forgeries.

### Evidence of mobility and connectivity on a spatial level: economic aspects

Neutron activation analysis can be extremely useful in establishing a high degree of mobility and connectivity. In terms of vessels, this can have different quantitative and spatial dimensions. For instance, certain wares were part of small-scale intra-regional movements. Jung (2021) explained how, in addition to Aegean imports, large storage vessels and Mycenaean-type and Mycenaeanizing ceramics produced on the Italian peninsula circulated between Apulia (Roca Vecchia, Caverna dell’Erba, and Scalo di Furno) and southern Calabria (Punta Zambrone) in the Late Bronze Age (see also Jung 2021a). However, the Italo-Mycenaean products circulated on a much smaller scale than the Aegean imports. This points to a network of small communities with independent exchange relationships along the shores of southern Italy. By contrast, workshops in some regions produced certain types of wares principally for export. The Hellespont workshops were a prime example (Fig. 2). During the archaic period, they manufactured pottery of the southern Ionian style (though not exclusively) for the Greek Apoikiai of the northern Black Sea coast (Posamentir & Solovyov 2006; Geissler et al. 2022, 240). A major production centre of eastern Greek-inspired pottery appears to have been located at Kelenderis in Cilicia (Lehmann 2021; Lehmann et al. 2020). According to recent NAA results, vessels decorated with bands were manufactured here from the fifth century BCE. Since such vessels, which were found throughout the Levantine region, fit the chemical fingerprint of Kelenderis, it can be assumed that they were made primarily for export—even though the site was not a major political player at the time. Without NAA, all examples cited would have remained undetected.

However, talking about mobility, it is not just about the pots but also about the potters. For instance, it has been shown that Late Bronze Age Grey Ware from Tiryns, a rare and ‘non-Mycenaean’ group in palatial and post-palatial assemblages reflecting Italic influence, was not imported but produced locally, using predominantly the chemically identical clay paste as for late-Mycenaean fine ceramics (Prillwitz 2021). The typological parallels between truly Italic and local Grey Ware are sufficiently close that immigrant Italian potters must be considered. Regarding the adoption of Mycenaean shapes and further chemical overlaps with Handmade Burnished Ware, the combination of formal, technological, and chemical observations suggests a strong interplay of local and foreign potting traditions.

### Evidence of cultural contacts and socio-cultural processes

Macroscopically foreign but chemically locally produced ceramics cannot be attributed to the efforts of branch workshops or migrating potters alone. In areas of pronounced inter-cultural contacts such as Iron Age southern Italy, Sicily, or southeastern Cilicia, distinct non-local influences can be discerned. The presence of mixed-style vessels in archaic western Sicily has been described as the result of a community of practice involving the appropriation and reshaping of external (Greek) elements for local purposes rather than direct physical contact between the producers and the recipients (i.e., Greeks, Phoenicians, and locals; Balco 2018). However, proximity to nodes of trans-regional (maritime) networks seems to be an important explanation for the extent and rate of acquisition of non-local influences, as Riehle (2021) demonstrated in a comparison of the ceramic production spectrum of three different sites in southern Italy and Sicily. Pottery production was already highly diversified in sixth century BCE Monte Iato in western Sicily, whereas more remote sites in the Apulian (Ascoli Satriano) and Lucanian (Ripacandida) hinterlands were much slower in responding to non-local (Greek) influences, if at all. These different responses of local potters can be linked to corresponding local demands and thus to varying social behaviour and development (Riehle et al. 2021a, b). Taking into account the found contexts, it is also possible to retrace the places of use of certain wares according to social practices. At Monte Iato, for example, the consumption of locally produced Greek and traditional pottery types within the sacred district was attributed to the multipolar expression of social identities by local elites. A similar pattern may be assumed for the sanctuary at Francavilla Marittima. Not least its proximity to the shores of northern Calabria had made it an important contact zone between local, Greek, and other actors since the Early Iron Age (for an archaeological overview see Jacobsen et al. 2018). Although NAA is still in its infancy here, local workshops were producing a highly diverse range of wares from a very early period, much of which was consumed within the religious area (Jacobsen 2021). Regarding the transfer of ceramic technology, NAA has highlighted new patterns in remote regions of the Balkan hinterland. A northern Aegean geometric ware (K-22 pottery) was produced using the same fabric comprising the same forms in microregions from the northern Aegean coast to the Balkan interior. A study of their cultural landscapes and social organisation has suggested a process of technology transmission of K-22 ware through demic rather than cultural diffusion (Gimatzidis 2021a).

## Analytical − methodological challenges and requirements

In addition to the technical aspects mentioned at the beginning of this paper, it is the experience gained over many years that makes NAA one of the most reliable instruments for the chemical determination of the origin of ceramics. However, recently, new demands have emerged, leading to modifications and refinements of procedures and applications. A process of continuous improvement by definition is never complete—especially when, as is the case with NAA, increasing quantities of data lead to the increasing differentiation of chemical groups. Therefore, it is extremely important to discuss both current difficulties and the strategic requirements for the successful application of the method.

### Problems and tackles

New challenges have emerged in sample series conducted in southern Italy (Jung 2021) and Sicily (Mommsen 2021), where (unlike the Levant, Greece, or the eastern Aegean) NAA has only recently been applied on a broader scale. Despite the high measurement precision, elemental concentration patterns of some ceramics are statistically hardly distinguishable, which must inevitably come from distant places. In some cases, there are also matches of raw clay and kiln waste samples from different sites, which raises the suspicion that this may be due to a strong homogeneity of trace element concentrations of certain deposits or their wide extension, whose clay(s) were used directly with no additional plastic components (Matricardi et al. 2021; Riehle et al. 2021a). Thus, a certain elemental concentration pattern can be determined as a fingerprint for a given site if suitable reference material has been sampled. Nevertheless, since this pattern can also be characteristic of other sites, local production spectra cannot be isolated or different sample series reliably compared. A similar phenomenon, albeit on a spatially smaller scale, can be observed for the so-called Mycenae-Berbati pattern, which appears to have been ‘produced’ at several sites in the northeastern Peloponnese (Mommsen 2012; Demakopoulou et al. 2017). Yet, certain vessel types exported to the Near East and Cyprus appear in quantity only at one of the hypothetical northeastern Peloponnesian production sites (namely, at Berbati), which narrows down the provenance of exports with that pattern (Jung 2015).

Such issues can be tackled at several levels. A possible approach, especially for regions where NAA is in its infancy (e.g., Italy) is to increase the number of samples from a given location. A larger quantity of samples enhances the chance of differentiating chemical groups and distinguishing between very similar clay pastes. In other words, increasing the number of samples enables a finer calibration of chemical groups. Raw clays and strong reference markers, such as misfired pieces and pottery waste, should be included whenever possible. Likewise, other analytical methods increasing the number of elements measured by NAA or including isotope concentrations can be considered. Petrographic analyses can also help to narrow down the region of origin of problematic or non-localizable chemical groups. By combining NAA with petrography, Ben-Shlomo et al. (2021) were able to identify Tell en-Nasbeh in the vicinity of Jerusalem and other regions outside Philistia as an Early Iron Age production site of Philistine Bichrome pottery. Another approach involves computer-based simulations of problematic group compositions or inter-group relations (Sterba 2021). Theoretical considerations regarding the cause of homogeneous elemental patterns, a large scattering of elemental concentration patterns, or the effects of different dilutions due to non-plastic additives can be verified, thus leading to a better understanding of the data.

### Strategic requirements

It is also necessary to consider strategic requirements. For individual projects and the research community in general, the fundamental aspects of sampling strategy and data publication must be discussed on an ongoing basis—regardless of the specific laboratory analysing the samples—if the benefits of NAA are to be fully exploited. For instance, reference material for the detection of local chemical fingerprints must be carefully selected. Even the classification of a sherd as a misfire (and therefore a strong local reference) may depend on very different parameters, which in worst-case scenarios can lead to incorrect localisations. Whether scattered finds of perhaps secondarily overfired pieces or the contents of a waste pit next to a pottery kiln are used can make the difference. Basically, of course, a very restrictive definition of a misfire should be aimed at, referring to unambiguous findings within a safely identified workshop. However, since unambiguous evidence is rare, it is even more important to make clear the composition, the find context(s), and the evaluation of the reference material. Lis (2021) drew attention to the importance of providing extensive coloured images and drawings for the publication of sampling series regardless of the localisation of chemical groups (e.g., Lis et al. 2020). Only then can the above-mentioned macroscopic feedback to non-sampled pieces be ensured, which in turn might lead to the refinement of the indispensable conventional visual classification of ceramics (see above). Publications could be supplemented by freely accessible online archives such as the Mendeley Data Repository. The same applies to the raw chemical data from each sample measured. However, to enable data comparison between sample series from different laboratories, details about other parameters must be made available, for example, the kind of drill bit that is used for sample extraction (usually Al_2_O_3_), the ceramic standard used, the mean elemental concentration values of the chemical groups detected (including their standard deviation), and the best-fit factor of the individual samples assigned to a group. As early as about 20 years ago, it was thus possible to link extensive data sets collected at Berkeley with the Bonn database (Mommsen et al. 2002).

One of the most important strategic requirements for researchers, especially archaeologists who are not familiar with the method, is a basic grasp of NAA. For instance, there can be misunderstandings about how to deal with results. This seems to be grounded in the different conceptions of natural sciences on the one hand and humanities on the other. For archaeology, NAA may be described as a dynamic tool. The precision of chemical group delineation and assignment of individual samples theoretically increases with each additional series of samples performed, which can lead to the assignment of a sample from the border area of one chemical group to another when new data are acquired. The sample composition of a chemical group may therefore change slightly. Similarly, a single group with a relatively large scatter in the elemental patterns of related samples could be broken down into two better-defined groups by further sampling. There is always the possibility that some assignments from published results may quickly go out of date. However, since this increases the reliability of the assignments of the core group, it is not a weakness but a strength of the method. For natural scientists, this may sound trivial, though it may raise the initial suspicion of archaeologists, who are accustomed to working with long-lasting typologies. Metaphorically speaking, the evaluation of a sample series should already resemble an interdisciplinary ping-pong match between archaeology and archaeometry, in which the ball passed from one side of the table is returned with a different spin. It is not least up to us to dispel any reservations through correspondingly transparent publications and to repeatedly emphasise the potential of NAA for archaeological research. This, after all, is a declared aim of our paper.

It would be remiss not to mention a fundamental problem that lies beyond the direct sphere of influence of archaeologists and archaeometrists already addressed by Tite (1999, 198)—the decline in research reactors offering NAA, particularly in Europe. This will lead not only to longer waiting times but also to increasing costs, as irradiated samples may need to be transported over long distances to measuring laboratories. It is an issue that affects not only archaeometry but any discipline engaged in research involving neutrons.

## Final remarks

Neutron activation analysis has been practised for more than 60 years. It remains one of the most reliable providers of proxy data for a broad range of archaeological concerns relating to the origin of pottery. Its various applications have been explained herein using old and recent examples. Starting with the simple question of where a vessel was made, NAA helps to confirm the mobility of materials and people, trace economic and/or social networks; provide insights into the social background of demand, production, and identity formation; and distinguish between originals and fakes. Beyond these advantages lies what is both a challenge and a strength: the need for intensive interdisciplinary collaboration, which, of course, is already embedded within the concept of archaeometry itself. However, unlike less labour-intensive methods of chemical provenance determination, NAA requires a tight interplay between scientists and laboratories because of the need for thorough sampling and measurement processes. Rather than taking this apparently more complex procedure to be a drawback, we argue that it ensures ongoing collaboration between the disciplines and a more ‘centralised’ practice, leading to the creation of larger, interregional, diachronic data banks (e.g., Bonn, etc.). In addition, and relatedly, only by re-evaluating older results and comparing them with new samples will complementary, revised processes of chemical group determination, and finer calibration of elemental concentration patterns be possible.

We hope that this discussion has successfully outlined the enormous benefits of NAA for colleagues who are occupied with the research questions raised herein. It is primarily up to those who are already familiar with NAA to keep demonstrating its strengths and to argue for its increased application. This is a vital step in enhancing and sustaining its reliability. The aforementioned issues will be clarified through its greater systematic (research-driven) use, which will in turn generate more accurate results. In addition, a certain degree of standardisation of procedures is needed, for example, regarding technical matters, sample strategies, terminology, or the publication of results. The FACEM database (https://facem.at), which is used to determine the fabric of ancient pottery, and the Levantine Ceramic Project (https://www.levantineceramics.org) provide excellent examples of such an approach. However, in arguing for more standardisation, we are not calling for everyone to follow a single uniform path, which in any case would be impossible. Any given research issue requires adaptation to specific local and/or situational circumstances. Yet, some standardisation is necessary if data are to be compared and used beyond a particular project and if conclusions are to be clearly understood. Otherwise, there is a danger that sample series will remain confined to the local, and this will make it impossible to frame them within a larger Mediterranean context. After all, the possibility that NAA can re-contextualise ancient pottery constitutes one of its great strengths—not just in the field of ceramic studies, but also for a deeper understanding of ancient societies and their cultural practices. Also, increased networking amongst researchers from different disciplines and projects and inter-laboratory cross-referencing of elemental concentration patterns are essential. In fact, a deeper understanding of ancient connectivity expressed through material culture requires at least as much connectivity between the disciplines studying it.

## Supplementary Information

Below is the link to the electronic supplementary material.

Supplementary file1 (DOCX 18 KB)

## Data Availability

Additional data (coordinates of the sites mentioned in the text) are openly available in the supplementary information section of Archaeological and Anthropological Sciences.
