# Supplementary material for: Neutron activation analysis in Mediterranean Archaeology: current applications and future perspectives
Source: Archaeol Anthropol Sci. 2023 Feb 11;15(3):25. doi: 10.1007/s12520-023-01728-1 (PMC9918394; doi:10.1007/s12520-023-01728-1)
Supplement: Supplementary file 1 — Supplementary file1 (DOCX 18 KB) [file 12520_2023_1728_MOESM1_ESM.docx]

**Neutron Activation Analysis in Mediterranean Archaeology: Current Applications and Future Perspectives**

**Supplement 1**

Geographical coordinates of the sites mentioned in the text:

| **Site** | **N** | **E** | **Referring to** |
| --- | --- | --- | --- |
| Ascoli Satriano | 41° 12′ 56.000“ | 15° 33′ 28.000“ | Modern town |
| Athens | 37° 58′ 40.000“ | 23° 43′ 40.000“ | Modern town |
| Berezan | 46° 35′ 58.000“ | 31° 24′ 41.000“ | Island |
| Caverna dell’Erba | 40° 19′ 29.000“ | 17° 44′ 48.000“ | Archaeological site |
| Clazomenae | 38° 21′ 29.400“ | 26° 46′ 03.300“ | Modern town (Urla) |
| Francavilla Marittima | 39° 48′ 22.000“ | 16° 22′ 27.000“ | Archaeological site |
| Hebron | 31° 31′ 57.000“ | 35° 05′ 59.000“ | Modern town |
| Jerusalem | 31° 46′ 45.000“ | 35° 13′ 25.000“ | Modern town |
| Kelenderis | 36° 08′ 37.000“ | 33° 19′ 22.000“ | Archaeological site |
| Kinet Höyuk | 36° 51′ 13.000“ | 36° 09′ 25.000“ | Archaeological site |
| Kyme | 38° 45′ 34.000“ | 26° 56′ 08.000“ | Archaeological site |
| Miletos | 37° 31′ 49.000“ | 27° 16′ 27.000“ | Archaeological site |
| Monte Iato | 37° 58′ 03.000“ | 13° 11′ 52.000“ | Archaeological site |
| Mykene | 37° 43′ 51.000“ | 22° 45′ 22.000“ | Archaeological site |
| Punta di Zambrone | 38° 41′ 53.000“ | 15° 59′ 30.000“ | Closest modern town |
| Ripacandida | 40° 54′ 46.000“ | 15° 43′ 32.000“ | Modern town |
| Roca Vecchia | 40° 17′ 14.000“ | 18° 25′ 34.000“ | Archaeological site |
| Scalo di Furno | 40° 16′ 11.000“ | 17° 52′ 50.000“ | Archaeological site |
| Tell en-Nasbeh | 31° 53′ 08.000“ | 35° 12′ 58.000“ | Archaeological site |
| Teos | 38° 10′ 46.000“ | 26° 46′ 59.000“ | Archaeological site |
| Tiryns | 37° 35′ 58.000“ | 22° 47′ 58.000“ | Archaeological site |
